# A qualitative investigation of the Montgomery–Åsberg depression rating scale: discrepancies in rater perceptions and data trends in remote assessments of rapid-acting antidepressants in treatment resistant depression

**DOI:** 10.3389/fpsyt.2024.1289630

**Published:** 2024-05-01

**Authors:** Gianna Capodilupo, Raymond Blattner, Anita Must, Silvia Gamazo Navarro, Mark Opler

**Affiliations:** ^1^ WCG Clinical Endpoint Solutions, Princeton, NJ, United States; ^2^ Seton Hall University, South Orange, NJ, United States; ^3^ Department of Psychiatry, Whanganui District Health Board, Whanganui, New Zealand; ^4^ Chelsea and Westminster Hospital NHS Foundation Trust, London, United Kingdom; ^5^ The PANSS Institute, New York, NY, United States

**Keywords:** depression, MADRS = Montgomery-Asberg depression rating scale, rater perception, rapid acting antidepressants, structured interview guide for the MADRS (SIGMA)

## Abstract

**Introduction:**

Despite the development of many successful pharmaceutical interventions, a significant subset of patients experience treatment-resistant depression (TRD). Ketamine and its derivatives constitute a novel therapeutic approach to treat TRD; however, standard tools, such as the Montgomery–Åsberg Depression Rating Scale (MADRS) are still being used to measure symptoms and track changes.

**Methods:**

The aim of this study was to review item-level differences between rate of data change (MADRS score) and rater-weighted perception of the most useful items for assessing change in symptoms while remotely conducting the 10-item version of the MADRS in TRD in a clinical trial of rapid-acting antidepressants. Two studies of rapid-acting antidepressants in the treatment of TRD were used to identify item-scoring trends when MADRS is administered remotely and repeatedly (733 subjects across 10 visits). Scoring trends were evaluated in tandem to a rater survey completed by 75 raters. This was completed to gain insight on MADRS items’ perceived level of helpfulness when assessing change of symptoms in rapid-acting antidepressant trials.

**Results:**

MADRS items ‘Reduced sleep’, ‘Apparent sadness’, and ‘Pessimistic thoughts’ were found to have the greatest average data change by visit, while raters ranked ‘Reported sadness’, ‘Lassitude’ and ‘Apparent sadness’ as the most helpful items when assessing symptom change.

**Discussion:**

The diversion between rate of data-change ranking and rater perception of helpfulness could be related to difficulty in assessing specific items, to the novel treatment itself, and/or to the sensitivity to symptom change to which raters are accustomed in traditional antidepressant treatments.

## 1 Introduction

In addressing the challenge of treatment-resistant depression (TRD), this manuscript focuses on the evolving landscape of depression treatment and the role of rapid-acting antidepressants (RAAD), such as ketamine and its derivative, esketamine. These novel treatments, approved by the FDA in 2019 for TRD (1), constitute a significant advance in the pharmacotherapy of depression, but pose new questions about the effectiveness of traditional assessment tools like the Montgomery–Åsberg Depression Rating Scale (MADRS) in evaluating rapid symptom changes. The 10-item MADRS, a standard in clinical research and practice, is used to measure symptom severity and changes in depressive syndromes, yet its suitability for novel, rapid-acting treatments remains under-explored, particularly in the context of remote administration (2, 3).

Depression, a leading contributor to global disability, affects about 322 million people worldwide, with an 18.4% increase in prevalence between 2005 and 2015 (4). Traditional treatments have evolved significantly since the 1950s, from tricyclic antidepressants and monoamine oxidase inhibitors to second-generation antidepressants like SSRIs and SNRIs. However, for a substantial subset of patients, these treatments fail to provide full or partial remission, leading to the classification of TRD (5–7). The complexity of defining and measuring treatment resistance, as highlighted by studies like the Sequenced Treatment Alternatives to Relieve Depression (STAR*D) trial, underscores the need for effective treatment options and reliable assessment methods (8–10). Rapid-acting antidepressants, a departure from the monoaminergic focus of traditional treatments, offer a promising avenue for treating TRD. Ketamine, antagonizing the NMDA receptor, induces a glutamate surge, fostering brain adaptability and pathway creation (11, 12). Its rapid symptom relief, observed within 24 to 72 hours post-administration, marks a stark contrast to the gradual effects of conventional antidepressants (13–15). However, the long-term effects and optimal dosing of ketamine remain areas for further research (16–18).

The MADRS, designed to sensitively capture treatment-induced symptom changes in depression, has historically been administered in-person but is increasingly used in remote settings. This shift raises concerns, particularly for items like ‘Apparent sadness,’ which rely on observational assessment (19–24). Despite its widespread use and proven interrater reliability across various languages, the appropriateness of MADRS for rapid-acting antidepressants, especially in remote settings, warrants examination (25–35). In addition to that RAADs can generate responses within hours or days, rather than weeks or months, while our rating instruments were designed to assess mood symptoms over a 7-day time frame, typically. Consequently, adaptative approaches are required for existing scales to meet the need of adjustment. Yavorsky et al. provide an excellent summary on adaptations to the standard rating instruments allowing to reflect short-term changes in which RAADs act, as well as implementing novel rating measures. Critically, they also discuss limitations and challenges to the currently used rating measures including any conceptual biases of raters (36).

This publication, therefore, seeks to analyze the item-level differences in MADRS scores and the raters’ perception of their helpfulness in determining symptom severity change in TRD during clinical trials of rapid-acting antidepressants. The study hypothesizes that while some MADRS items may align with rater perceptions, discrepancies are expected due to the unique nature of rapid-acting antidepressant treatment and the challenges of remote assessment. This investigation is crucial for ensuring that depression assessment tools remain relevant and effective in the rapidly evolving landscape of antidepressant therapy.

## 2 Materials and methods

### 2.1 Data change on MADRS items in two protocols of a phase 3 study

We have established the rate of change of all items of MADRS for the entirety of two separate protocols of a Phase 3 study of rapid-acting antidepressants using a similar design, but varying in terms visit frequency (between 24 hours and 7 days). We used deidentified datasets only containing the visit date, but no other information that would be considered protected health information, only to perform a qualitative, exploratory analysis looking at individual MADRS items and changes in their score across visits. Rate of change was calculated by first determining between-visit MADRS data change by item; we then divided the per-item change by the number of days that had passed between an individual visit and the previous visit to establish change per day. We rank-ordered the per-visit change for a given item. The data was solely used to create the rankings discussed, and no other quantitative analysis has been completed for the purpose of this study.

### 2.2 Remote rater-experience survey

We conducted a survey of clinical research professionals who have participated in the above-mentioned Phase 3 programs of rapid-acting antidepressants utilizing new modalities for the assessment of depressive symptoms, including remote evaluation technologies (telepsychiatry) and versions of MADRS that have been adapted for use over shorter recall periods, e.g. last 24 hours. A total of 75 experienced raters from 13 countries were recruited for this survey study, all of whom participated in at least one TRD clinical trial for rapid-acting antidepressants. All survey participants were polled about which MADRS items they considered most and least helpful for assessing changes in symptoms in two protocols of a Phase 3 study with the same rapid-acting agent to treat TRD. Many raters participated in these studies on almost a daily basis, with a rotation of subjects. Items considered as most useful as endorsed by raters for assessing TRD related symptom changes were ranked as most important, second most important, and so on.

We compared the data change on MADRS items and the raters’ rankings to gain additional insight into a.) the rater experience of each item’s helpfulness in determining depression severity and b.) the rate of change in MADRS scores in a clinical trial for rapid-acting antidepressants in TRD.

## 3 Results

**Figure 1:**
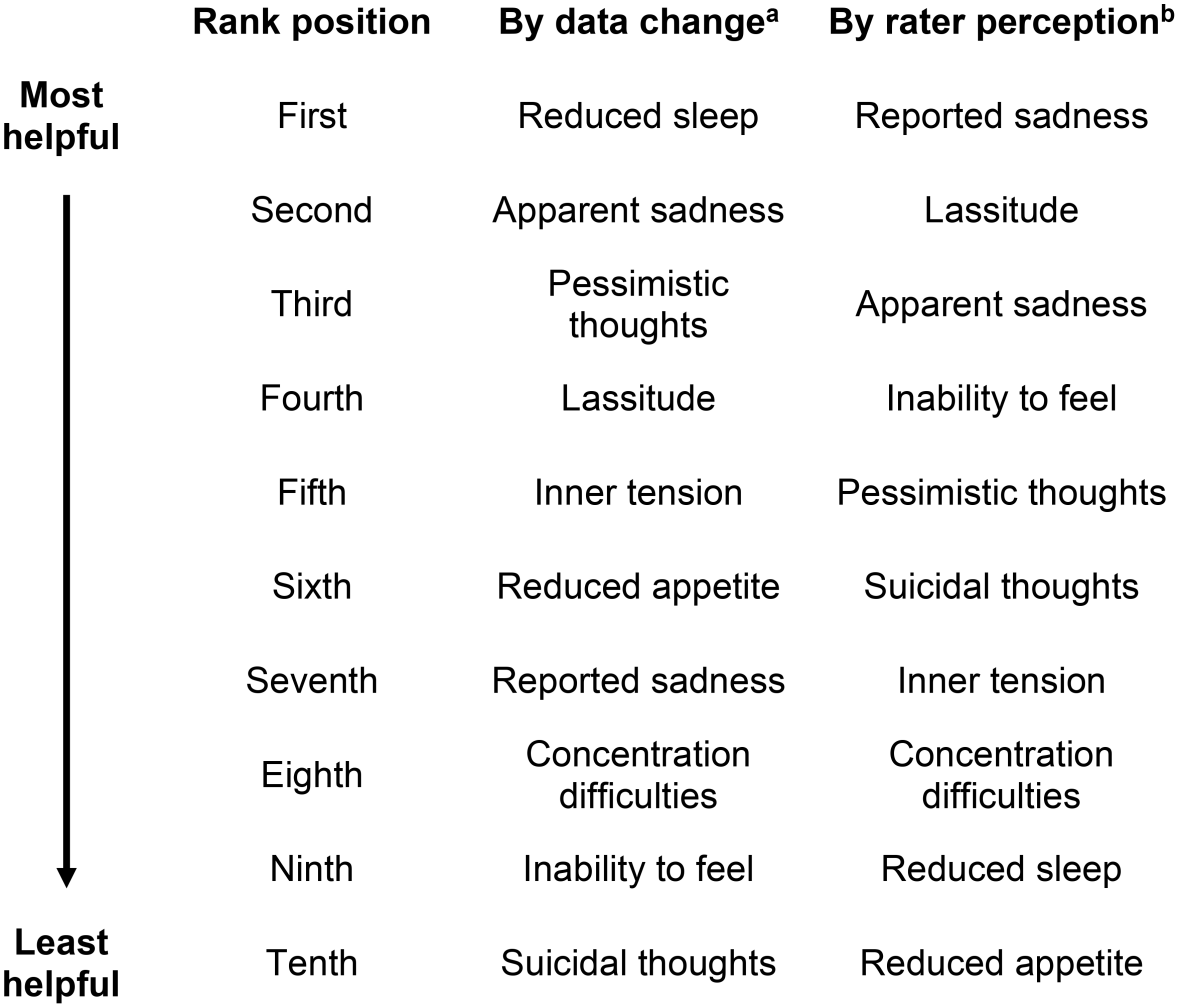
Ranking of MADRS items according to rater perception and data change. ^a^Ranking by data change: ranking from tenth position to first position correlates with the average rate of data change between visits, with first position representing the MADRS item with the highest rate of symptom change according to data. ^b^Ranking by rater perception: ranking from tenth position to first position correlates with the degree of helpfulness in assessing symptoms of depression, with first position representing the MADRS item perceived as the most helpful when assessing symptom change.

Surveyed raters conducted assessments via telephone to ensure the integrity of blinding in these trials. Raters were selected from studies with more than 700 subjects combined (N = 733) across 20 countries and 16 different languages. In the two examined protocols of a Phase 3 study with the same rapid-acting agent to treat TRD, 733 subjects were included in the dataset across 10 visits (excluding early termination; 12 in visits in total). MADRS total scores ranged from 0 to 53 across visits for each protocol. Mean total MADRS score was 30.39. Individual item scores ranged from 0 to 6 for each item. Ranking of items was determined by rater perception of helpfulness in assessing symptom change, and by average MADRS score change in between-visit data. Rater ranking order (from 10 to 1) correlates with an increasing level of perceived helpfulness in determining symptom change, with 1 being the most helpful. Data ranking of MADRS items based on the deidentified dataset (from 10 to 1) correlates with a greater average rate of MADRS item-level score change between visits, with 1 representing the item with the greatest average between-visit MADRS item score change. Helpfulness of MADRS items in assessing symptom change, ranked according to average rate of data change and by rater perception of helpfulness is shown in **Figure 1**, with a ranking in the first position representing the MADRS item with either the highest rate of symptom change according to greatest average between-visit MADRS data change (‘By data change’) or as being perceived as the most helpful by raters (‘By rater perception’) when assessing symptoms.

‘Reduced sleep’, ‘Apparent sadness’, and ‘Pessimistic thoughts’ were found to have the highest rate of data change by visit, however raters ranked ‘Reduced sleep’ in the ninth position of helpfulness, with ‘Apparent sadness’ and ‘Pessimistic thoughts’ placed third and fifth in terms of perceived helpfulness. In contrast, ‘Reported sadness’ was perceived as being the most helpful item for raters in assessing symptoms of depression but ranked seventh according to rate of data change. ‘Reduced appetite’ was ranked as the least useful item by rater perception, while rate of data change places this in the sixth position of helpfulness. ‘Suicidal thoughts’ was ranked sixth according to rater perception but placed tenth by rate of data change. The low ranking according to data change was caused by the smallest average change between visits for this item. ‘Inability to feel’ is ranked as the fourth most helpful item in terms of rater perception and shows the second lowest average change between visits in the data, ranking ninth according to data change.

## 4 Discussion

When examining the first item of the MADRS, ‘Reported sadness’, the account of symptoms and depression by subjects is the most important evidence one can receive. However, several factors including age of subject, age at onset of first depressive episode, education, length of illness, etc. could influence how sadness is being reported by subjects (37), and these may be due to differences in how depression has been conceptualized (38). Therefore, relying heavily on clinical background can be imperative for the clinician when administering and scoring the MADRS. ‘Apparent sadness’ also appears to be of critical interest, as it would have been presumed as a difficult item to assess in remote assessments. Nevertheless, both earlier studies by Kobak et al. (32) and a recent study by Sumiyoshi et al. conducted in Japan (39) found excellent reliability for the MADRS in remotely interviewing patients with MDD showing high consistency between remote and in-person interviews. These studies also emphasize the key importance of well-trained raters, which might be critical for accurately assigning ratings for more challenging items such as ‘apparent sadness’.

Appetite changes are not shared symptoms across all subjects with depression, and there can be marked increases or decreases in appetite in patients diagnosed with depression. Simmons et al. (40) report that only 48% of subjects experience reduced appetite, leaving 52% unaccounted for. Raters may be noticing this disconnect, with the focus of appetite in MADRS relying on reduction as opposed to bidirectional change and weighting the importance of this item accordingly.

We believe the discrepancy between rater-perceived ranking and ranking based on rate of data change for ‘Reduced sleep’ is due to the variability in the data itself. Sleep as a construct may be moving too quickly to be meaningful to raters more used to conducting traditional assessments. Additionally, sleep can move in more than one direction (i.e., it can both increase and decrease), and can be attributed to multiple factors not including symptoms of depression or drug treatment effects (environmental, pain, change in caffeine consumption, etc.), potentially deprioritizing sleep to raters and encouraging them to rely more heavily on other items.

Ranking for raters on ‘Suicidal thoughts’ (6^th^ on perceived helpfulness, but last on data change order) could demonstrate the importance of this item when it is reported, although the frequency of report may be low, and overall severity in most cases may also be low. When patients report suicidal thoughts or behaviors during MADRS interviews, the weight of this item may increase for raters, although the frequency with which it is seen in our dataset was also low.

In assessing depression, ‘Inability to feel’, involving changes in emotions, may become one of the most useful items to assess overall depression when reported, however, it may again be an item that infrequently shows large variability over shorter assessment periods.

The performance of traditional assessments to obtain ratings/scores for studies of novel treatments for depression is a critically important matter. It is imperative to reliably measure changes in symptoms—particularly for treatments that may carry unique side-effect profiles and safety risks. Researchers have used assessments on the widely agreed upon core symptoms of depression for decades. MADRS, though widely accepted by regulatory agencies and used by clinicians and researchers, may have a different value and clinical significance in the context of rapid-acting antidepressants. Using MADRS in short-interval, remote evaluations, with repeated assessments performed within 24 hours, might present challenges in accurately capturing symptom change. Certainly, not all depressed individuals have the same depressive symptoms at baseline and the dynamic nature of a therapeutic response to RAADs could potentially result in a rapid alleviation of certain symptoms, e.g. improvements in subjective mood, while leaving some other functional aspects less improved, resulting in a heterogeneity of symptom resolution and a sense of uncertainty in raters (36).

As research increasingly supports the use of rapid-acting antidepressants, and as their market approval increases, it is incumbent on clinicians to review and refine assessment processes. The rapid change in symptoms presents a challenge for clinicians, especially if the assessment is administered remotely. This study has compared how the rate of between-visit data changes for MADRS items relate to those that raters have identified as being more helpful or more challenging. The diversion between rate of data-change and rater perception of helpfulness could be related to several factors, one being difficulty in assessing specific items. A depression rater training study by Sajatovic et al. (41) showed no significant difference between raters based on country, level of experience with diagnosis, or previous training in terms of the items they identified as the most difficult to rate, namely ‘Apparent sadness’, ‘Inner tension’, ‘Concentration difficulties’, ‘Lassitude’, and ‘Inability to feel’. Similar results have been shown when comparing rater training using MADRS to other mood rating scales, such as the Hamilton Depression Rating Scale (HAM-D) and Young Mania Rating Scale (YMRS) in a bipolar disorder trial (42). We see the same MADRS items (listed above) identified as most difficult to rate with no significant difference for raters based on country, experience, diagnosis, or previous training, suggesting the items themselves present difficulty to raters (43). MADRS has also been noted as a more difficult scale to utilize when compared to other commonly used depressive symptom rating scales (HAM-D and YMRS), thus it could benefit from further insight and qualitative analysis (41).

A recent factor analysis conducted on two esketamine trials has taken an interesting approach to explore potential symptomatic clusters grouped around the rate of symptom change as detected by MADRS items (44). Three factors were identified labelled as affective/anhedonic symptoms (apparent sadness, reported sadness, lassitude, inability to feel); anxiety and vegetative symptoms (inner tension, reduced sleep, reduced appetite, concentration difficulties); and hopelessness (pessimistic thoughts, suicidal thoughts). Strikingly, our results on rater perception of items follows exactly these clusters with the affective/anhedonic factor listed as the most helpful for raters, followed by the hopelessness factor, and lastly the anxiety and vegetative factor symptom group, which is probably prone to the highest variability and heterogeneity overall.

Thus, another aspect potentially explaining the diversion between MADRS score changes and raters’ perceptions could be the novel treatment itself or the sensitivity to the consequent symptom change to which raters are accustomed. An example for this is the fluctuation of sleep on a day-to-day basis that can affect daily ratings when assessing change in symptoms with the MADRS. Research indicates that MADRS item responses - those related to sleep, most specifically - change in relation to patient experience and when compared to the prior administered assessment. Sleep, however, also influences memory and emotional memory in subjects with depression (45) and this can present additional challenges when ratings are conducted based on subject’s report every day or every other day, as is common in clinical trials of rapid-acting antidepressants.

There are inherent complications in the assessment of rapid change in TRD over short periods of time and rapid (i.e., between-visit) symptom changes at the item level can pose a challenge to ranking and assessing for severity. Singh et al. (14) examined the efficacy of both full and abbreviated MADRS scores in evaluating the response to intravenous esketamine. They conducted assessments of depressive symptoms at 2-, 4-, and 24-hours post-infusion, opting to omit the sleep and appetite items for the shorter 2 and 4-hour assessments. Johnson and colleagues also investigated the MADRS’s suitability within a 24-hour recall period, finding comparable content validity and high internal consistency and test-retest reliability (46). While most participants deemed a 24-hour recall period sufficient for assessing meaningful changes in depression symptoms, reduced sleep and appetite were noted exceptions, echoing Singh et al.’s decision to exclude these items. Yavorsky et al. analyzed esketamine trial data, revealing the MADRS’s sensitivity to change over short periods, albeit with limited responsiveness in the sleep and suicide items (47). These findings underscore the challenge of effectively capturing clinical change within a 24-hour timeframe, particularly for traditional depressive symptoms like mood, appetite, and sleep disturbances. Novel treatments for TRD have yet to yield novel assessments that are sensitive to change over such short periods of time.

Each item discussed and present in the MADRS is important in assessing overall depression and symptomology of the diagnoses, and clinical experience brings clarity to differences in items and scores, such as the importance of suicidal thoughts and sleep deprivation.

## 5 Conclusion

Rapid-acting antidepressants, such as ketamine and its derivatives, appear to induce more rapid changes in depression symptoms, which presents a challenge in the accurate capture of symptom change when using conventional rating scales. Our findings indicate a diversion between the rate of data change as measured by MADRS scores vs the raters’ perception of helpfulness of specific MADRS items in determining clinical improvement and depression severity change. While each MADRS item remains important in an assessment of depression symptomatology, it may be beneficial to refine raters’ sensitivity to changes in depressive symptoms over short periods of time, and to the specific side effects associated with novel treatment approaches. Further to this, gaining experience in the use of rapid-acting antidepressants, and in the ability to measure short and long-term effects of these novel treatments, might influence our notion of defining criteria for treatment resistance in depression.

## Data availability statement

The raw data supporting the conclusions of this article will be made available by the authors, without undue reservation.

## Ethics statement

Ethical review and approval was not required for the study of human participants in accordance with the local legislation and institutional requirements. Written informed consent from the participants was not required to participate in this study in accordance with the national legislation and the institutional requirements.

## Author contributions

GC: Writing – review & editing, Writing – original draft. RB: Writing – review & editing, Writing – original draft. AM: Writing – review & editing, Writing – original draft. SN: Writing – review & editing, Writing – original draft. MO: Writing – review & editing, Writing – original draft.
